# N-acetylglutamic acid alleviates oxidative stress based on histone acetylation in plants

**DOI:** 10.3389/fpls.2023.1165646

**Published:** 2023-05-08

**Authors:** Takeshi Hirakawa, Seia Tanno, Kazuaki Ohara

**Affiliations:** Kirin Central Research Institute, Kirin Holdings Company, Ltd., Fujisawa, Kanagawa, Japan

**Keywords:** N-acetylglutamic acid, chemical priming, oxidative stress, epigenetic regulation, histone acetylation

## Abstract

Oxidative stress causes cellular damage and genomic instability through the accumulation of reactive oxygen species (ROS) in plants, resulting in reduced crop production. Chemical priming, which can enhance plant tolerance to environmental stress using functional chemical compounds, is expected to improve agricultural yield in various plants without genetic engineering. In the present study, we revealed that non-proteogenic amino acid N-acetylglutamic acid (NAG) can alleviate oxidative stress damage in *Arabidopsis thaliana* (Arabidopsis) and *Oryza sativa* (rice). Exogenous treatment with NAG prevented chlorophyll reduction induced by oxidative stress. The expression levels of *ZAT10* and *ZAT12*, which are regarded as master transcriptional regulators in response to oxidative stress, increased following NAG treatment. Additionally, Arabidopsis plants treated with NAG showed enhanced levels of histone H4 acetylation at *ZAT10* and *ZAT12* with the induction of histone acetyltransferase*s HAC1* and *HAC12*. The results suggest that NAG could enhance tolerance to oxidative stress through epigenetic modifications and contribute to the improvement of crop production in a wide variety of plants under environmental stress.

## 1 Introduction

As sessile organisms, plants are exposed to various environmental stress factors, such as heat, drought, cold, and salinity, which inhibit growth and reduce crop biomass. In response to environmental stress in plants, oxidative stress, which is caused by the accumulation of reactive oxygen species (ROS) derived from electron energy in the chloroplasts, mitochondria, and peroxisomes, causes cellular damage through membrane oxidation and the collapse of photosynthesis. ROS generation is induced by both biotic and abiotic stresses, such as microbial pathogens (Kim and Hwang, 2014; Yang et al., 2017). Abiotic and biotic stresses disrupt the equilibrium between production and removal of intracellular ROS. This disruption of ROS equilibrium leads the overaccumulation of ROS, which causes oxidative stress. Oxidative stress also threatens the genome stability of plants owing to genotoxic stress, leading to effects such as replication error, cell cycle arrest, and cell death (Baxter et al., 2014). Therefore, plants must respond rapidly and plastically to oxidative stress to maintain their stability at the cellular and genomic levels. In response to plant oxidative stress, low molecular antioxidants, such as flavonoids, anthocyanin, ascorbate and glutathione, are accumulated in tissues, therefore preventing cellular damage caused by ROS (Gechev et al., 2006). Arabidopsis overexpressing flavonol regulator *MYB12/PEG1* and anthocyanin regulator *MYB75/PAP1* accumulates flavonoids and anthocyanins, such as glycosides of kaempferol, quercetin, and cyanidin, which increases its tolerance to oxidative and drought stress (Nakabayashi et al., 2014). In addition to non-enzymatic ROS scavenging systems, ROS is removed by several antioxidant enzymes in plants. Superoxide anion ( ${\text{O}}_{2}^{\;\cdot -}$ ) generated in chloroplasts is altered to O_2_ or H_2_O_2_ by superoxide dismutases (SODs), and then H_2_O_2_ is detoxified by ascorbate peroxidases (APXs) to maintain photosynthesis activity, under stress conditions (Alscher et al., 2002; Shigeoka et al., 2002). Conversely, in mitochondria, the detoxification of H_2_O_2_ by glutathione peroxidases is achieved to maintain cellular respiration, suggesting that genetic engineering to improve the expression of such antioxidant enzymes in plants confers tolerance to oxidative stress (Anjum et al., 2016). Overexpression of *Populus ascorbate peroxidase* (*PpAPX*) in *Nicotiana tabacum* alleviated not only oxidative stress but also salinity and drought stress through increases in chlorophyll content and APX activity (Li et al., 2009). Additionally, *Ipomoea batatas* overexpression of *lbAPX* results in the acquisition of tolerance against salinity stress, with a reduction in ROS accumulation (Yan et al., 2016).

In addition to genetic engineering, chemical priming, which is a strategy that enhances tolerance to environmental stress based on the regulation of gene expression using functional chemical compounds, is expected to improve crop production in a wide range of plant species (Sako et al., 2021). Exogenous treatment with acetic acid increases the expression levels of jasmonate signalling pathway genes, conferring tolerance to drought stress in several plant species, such as *Arabidopsis thaliana* (Arabidopsis), *Zea mays*, *Triticum aestivum*, *Oryza sativa* (rice), and *Brassica napus* (Kim et al., 2017). Arabidopsis and *Lactuca sativa* plants treated with ethanol exhibit enhanced tolerance to heat stress, which is associated with the stimulation of response to unfolded proteins (Matsui et al., 2022). Heat stress is also modulated following treatment with β-aminobutyric acid (BABA) in Arabidopsis and *Brassica rapa*, through the induction of heat shock proteins and the improvement of photosynthesis performance and antioxidant systems, respectively (Zimmerli et al., 2008; Quan et al., 2022). Although chemical priming is an effective strategy of alleviating damage caused by environmental stress in plants without genetic engineering, little is known about the chemical compounds that enhance tolerance to oxidative stress in plants.

Studies using *Saccharomyces cerevisiae* (yeast) have shown that arginine metabolism plays a role in protection against damage caused by oxidative stress. *N*-acetyltransferase *MPR1*, which converts proline metabolism intermediate Δ^1^-pyrroline-5-carboxylate (P5C)/glutamate-γ-semialdehyde (GSA) to *N*-acetyl-GSA in arginine metabolism, is required for the alleviation of oxidative and heat stress in yeast (Nishimura et al., 2010). *MPR1* confers tolerance to these stressors by preventing ROS accumulation associated with arginine synthesis. Furthermore, nitric oxide (NO) converted from arginine by the flavoprotein Tah-18 contributes to stress tolerance in yeast suggesting that the enhancement of arginine metabolism could confer tolerance to oxidative stress in plants (Nishimura et al., 2013). In fact, overexpression Solanum lycopersicum *N-acetylglutamate synthase1* (*SlNAGS1*), which converts glutamic acid (Glu) to N-acetylglutamic acid (NAG) in the first step of arginine metabolism in chlorophyll, alleviates the damage caused by drought and salt stress through the accumulation of intermediates of arginine metabolism in Arabidopsis (Kalamaki et al., 2009). However, little is known about the effects and the underlying molecular mechanisms of arginine metabolism intermediates during chemical priming.

The expression of stress-responsive genes in plants is plastically and rapidly regulated by histone modifications, especially histone acetylation which is closely related to transcriptional activation (Sako et al., 2021). Histone H3 lysine 9 acetylation (H3K9ac) and H4 acetylation (H4ac) increase Arabidopsis T87 cell and tobacco BY-2 cell in response to cold and salinity stress (Sokol et al., 2007; Kurita et al., 2017). Deficiency in HDA9, which is one of the histone deacetylases, results in reduction of heat stress tolerance due to deposition of H3K9ac at heat stress-responsive genes in Arabidopsis (Niu et al., 2022). Additionally, the inhibitor of class I-type histone deacetylase (HDAC) alleviates salinity stress in Arabidopsis by upregulation of Na^+^/H^+^ antiporter *SOS1*, in response to the activation of H4ac (Sako et al., 2016). These findings suggest that the fine-tuning of histone acetylation is important for plant response to various environmental stress.

In the present study, we revealed that the non-proteogenic amino acid N-acetylglutamic acid (NAG), an intermediate in arginine metabolism in plants, can be used for chemical priming against oxidative stress. In Arabidopsis, exogenous treatment with NAG conferred tolerance to oxidative stress through the upregulation of oxidative stress-responsive genes, with an increase in histone acetylation levels. Additionally, rice treated with NAG exhibited tolerance to oxidative stress, based on the elevated expression levels of antioxidant enzymes, indicating that NAG could be used as a priming compound to enhance tolerance to oxidative stress in plants.

## 2 Materials and methods

### 2.1 Plant materials and growth condition

Arabidopsis plants used in the present study were Col-0 accessions. Sterilized Arabidopsis seeds were incubated in D.W. at 4°C for 24 h. After incubation, seeds were sown in liquid medium containing 1/2 Murashige and Skoog (MS) medium and 1% (w/v) sucrose, and then placed in an incubator set at 22°C, with a 16-h light/8-h dark photoperiod. Rice plants used in the present study were Nipponbare cultivar. Sterilized rice seeds were incubated in liquid medium containing 1/2 MS in an incubator set at 30°C, with a 16-h light/8-h dark photoperiod.

### 2.2 Measurement of chlorophyll content

Chlorophyll content was measured as previously described (Yamaguchi et al., 2021). To measure Arabidopsis chlorophyll contents, seven-day-old seedlings were treated with 0–0.4 mM N-acetylglutamic acid (NAG, Tokyo Chemical Industry Co., Ltd.), under 5 μM methyl viologen (MV, Sigma-Aldrich), for 48 h. Five seedlings were placed in 1 mL *N, N*′- dimethylformamide (DMF) in 1.5-mL tubes, and then incubated at 4°C for 24 h. After incubation, the absorbance of the extraction liquid was measured at 646.8 nm and 663.8 nm on an NP80 spectrophotometer (Implen, Munich, Germany). Total chlorophyll contents were calculated using the following formula: Chl a + b (μM) = 19.43 *A*_646.8 +_ 8.05 *A*_663.8_. To measure the chlorophyll content of rice, three-day-old plants were treated with NAG (0.5 mM) for 24 h and then incubated with 2.5 mM MV for 48 h. Three second foliage leaves were placed in 1 mL DMF. After incubation at 4°C for 24 h, the extraction liquid was used as a sample for the measurement of chlorophyll content. Each chlorophyll content (μM/mgFW) was determined relative to the chlorophyll content of plants with 0 mM NAG and 0 μM MV.

### 2.3 DAB staining

DAB staining was carried out as previously described with minor modification (Yamaguchi et al., 2021). Seven-day-old Arabidopsis seedlings were treated with 0.4 mM NAG and 5 µM MV for 24 h. After incubation, seedlings were stained with peroxidase stain DAB Kit (Nacalai) for 2 h in dark with gentle shaking. Stained seedlings were transferred to bleaching solution (60% ethanol, 20% acetic acid and 20% glycerol) and boiled for 15 min. Samples mounted with 50% glycerol were observed with SZX16 (EVIDENT) equipped with DP23 digital camera (EVIDENT). The experiment was conducted using three biological replicates.

### 2.4 RNA extraction and gene expression analysis

Total RNA was isolated from whole seedlings of Arabidopsis and rice using an RNeasy Plant Mini Kit (QIAGEN, Hilden, Germany). RNA was extracted according to the manufacturer’s instructions. Genomic DNA was extracted using an RNase-free DNase set (QIAGEN).

For gene expression analysis, cDNA was synthesized from 1 µg total RNA with a Verso cDNA Synthesis Kit (Themo Fisher Scientific, Waltham, MA, USA). RT-qPCR analysis was performed using TB Green *Premix EX Taq* II (TaKaRa Bio Inc., Shiga, Japan), gene-specific primers, and a Light Cycler 480 (Roche, Basel, Switzerland). *PP2AA3* and *OsACTIN1* (gene annotation: Os03t0718100) were used as internal controls in Arabidopsis and rice, respectively. Normalized expression levels were calculated using 2^(-delta delta Ct) method. The primers used for the RT-qPCR analyses are listed in **Supplementary Table S1**.

### 2.5 Chromatin immunoprecipitation and qPCR

Chromatin immunoprecipitation (ChIP) was performed as previously described, with minor modifications (Yamaguchi et al., 2014). Seven-day-old seedlings were treated with 0.4 mM NAG for 2 h and fixed with 1% formaldehyde for 15 min. After neutralization of the formaldehyde with glycine for 5 min, 1 g of the tissue was ground in liquid nitrogen using a mortar and pestle. Chromatin was isolated from the nuclear extract using nuclear extraction buffer. Fragmentation of chromatins were performed with Bioruptor^®^ UCD-250 (Cosmo Bio., Ltd., Tokyo, Japan) using power mode H and an on/off cycle of 30 s/60 s, for a total duration 12 min, on ice. Antibodies were added to the fraction of fragmented chromatins after preclearing, and then the samples were rotated overnight at 4°C. Five microliters of anti-H4ac antibody (Merck Millipore: 06-866) and 2 µL of anti-H3K4me3 (Abcam: ab8580) were used in immunoprecipitation.

qPCR analysis was performed using TB Green *Premix EX Taq* II (TaKaRa), gene-specific primers, and a Light Cycler 480 (Roche). *TA3* was used as the negative control. The primers used for the qPCR analysis are listed in **Supplementary Table S1**. ChIP experiments were repeated three times with three technical replicates. Statistical analysis was performed using Student’s *t*-test.

## 3 Results

### 3.1 N-acetylglutamic acid alleviates oxidative stress in *Arabidopsis thaliana*

**Figure 1:**
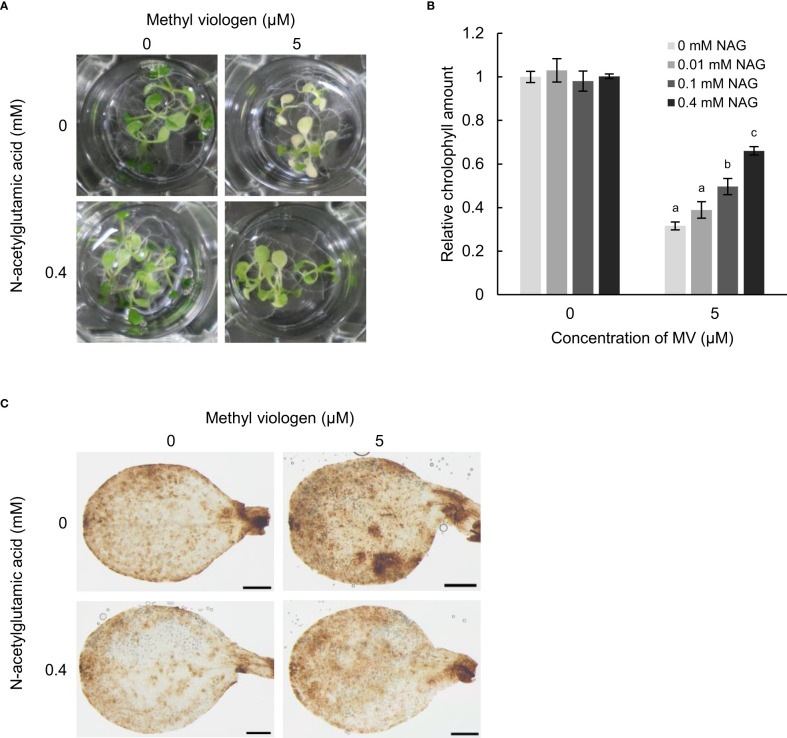
N-acetylglutamic acid enhances oxidative stress tolerance in *Arabidopsis thaliana*. **(A)** Seedlings treated with 0, 0.01, 0.1, and 0.4 mM N-acetylglutamic acid (NAG) under 5 μM methyl viologen (MV) for 48 h. **(B)** Chlorophyll contents in seedlings treated with 0, 0.01, 0.1, and 0.4 mM NAG under 5 μM MV for 48 h. Error bars indicate standard error. *n* = 15. *P* < 0.05 (Tukey test). **(C)** DAB staining for the detection of H_2_O_2_ in cotyledons of seedlings treated with or without 0.4 mM NAG and 5 μM MV for 24 h. Three biological replicates were conducted. Scale bar: 0.5 mm.

To confirm whether NAG has a capacity to alleviate oxidative stress damage in plants, we observed the responses of Arabidopsis to oxidative stress following treatment with NAG. Arabidopsis plants treated with MV, which produces reactive oxygen species (ROS) in chloroplasts, and is used to induce oxidative stress in plants, showed chlorosis of the cotyledons with a reduction in chlorophyll content (**Figures 1A, B**). In contrast, MV-induced chlorosis and reduction in chlorophyll content were suppressed in plants treated with NAG, in a dose-dependent manner (**Figures 1A, B**). DAB staining, which could detect H_2_O_2_ as a brown signal in tissues, also showed that the frequency of MV-induced H_2_O_2_ in seedlings treated with NAG was lower than that of seedlings without NAG (**Figure 1C**). These results suggest that NAG alleviated oxidative stress by preventing chlorosis and the accumulation of ROS in Arabidopsis.

### 3.2 N-acetylglutamic acid enhances the expression of genes involved in responses to oxidative stress

**Figure 2:**
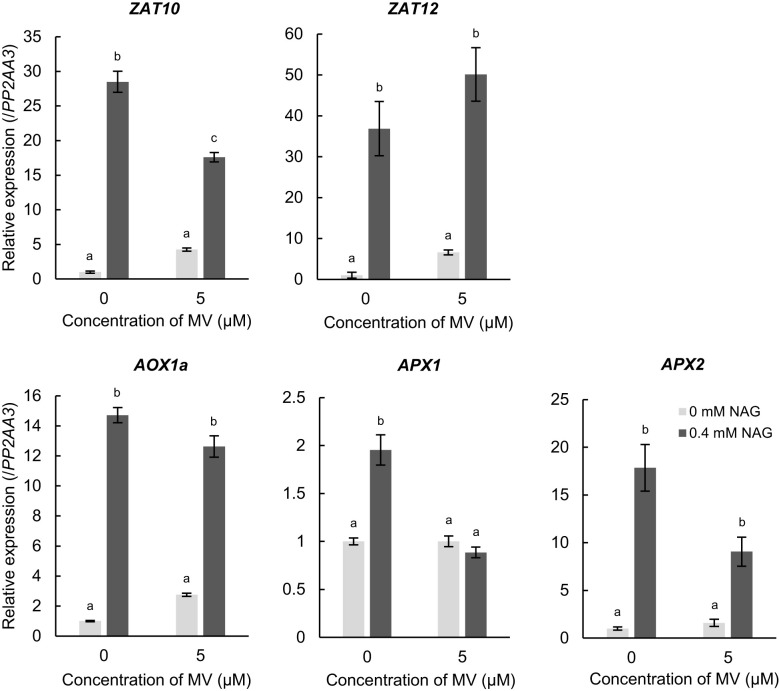
Expression levels of genes involved in responses to oxidative stress increased following treatment with N-acetylglutamic acid (NAG). Expression levels of *ZAT10*, *ZAT12*, *AOX1a*, *APX1* and *APX2* in seedlings treated with or without 0.4 mM NAG and 5 μM methyl viologen for 2 h. Error bars indicate the standard error. *n* = 3. *P* < 0.05 (Tukey test).

Several amino acids alleviate environmental stress in plants by activating the expression of stress-response genes. Therefore, we investigated whether NAG activates the expression of genes involved in response to oxidative stress in Arabidopsis. The expression levels of *ZAT10* and *ZAT12*, which are zinc-finger type transcription factors regarded as master regulators in response to oxidative stress in plants, increased with NAG treatment without MV (**Figure 2**). Furthermore, we observed that exogenous treatment of NAG increased the expression of *alternative oxidase 1a* (*AOX1a*) and *l-ascorbate peroxidase1* and *2* (*APX1* and *APX2*), which scavenge ROS and modulate cellular damage in response to oxidative stress (**Figure 2**). Induction of the genes above was observed in plants treated with NAG under control conditions, which was unrelated to MV treatment. According to the results, NAG increased the expression levels of oxidative stress-response genes, independent of oxidative stress damage.

### 3.3 Histone acetylation of *ZAT10* and *ZAT12* increased by N-acetylglutamic acid

**Figure 3:**
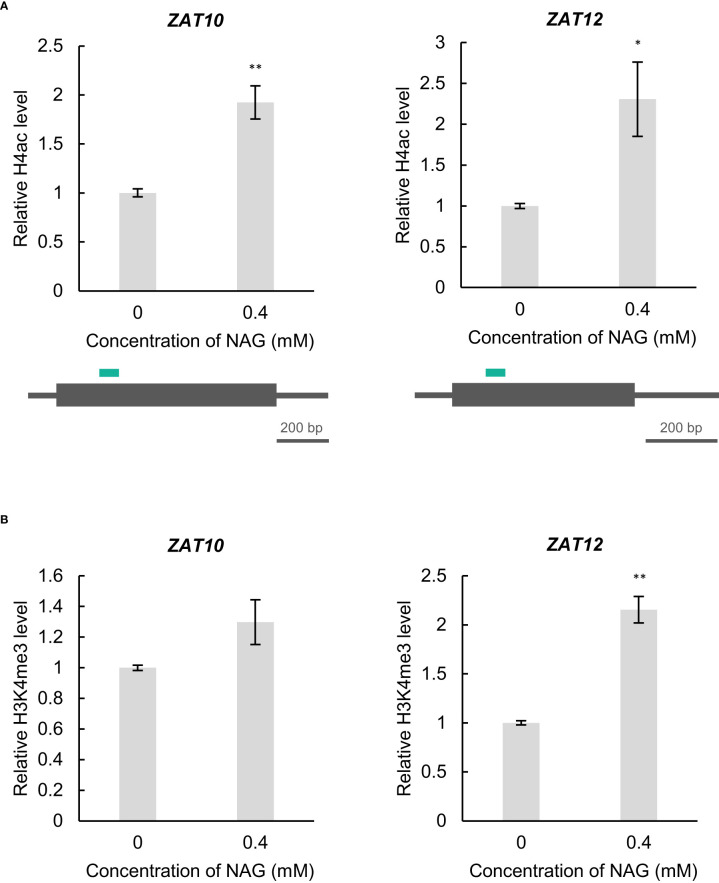
N-acetylglutamic acid (NAG) increases histone acetylation levels at *ZAT10* and *ZAT12.* **(A)** Top: Levels of histone H4 acetylation (H4ac) at *ZAT10* and *ZAT12* of seedlings treated with 0.4 mM NAG for 2 h. Error bars indicate standard error. *n* = 3. **P <0.01, *P < 0.05 (student’s *t*-test). Bottom: Diagrams of ChIP-qPCR analysis. Green bars indicate the regions amplified by qPCR. **(B)** Levels of histone H3 lysine 4 trimethylation (H3K4me3) at *ZAT10* and *ZAT12* in seedlings treated with 0.4 mM NAG for 2 h. Error bars indicate standard error. *n* = 3. **P < 0.01 (student’s *t*-test).

Phenotype and gene expression analyses revealed that NAG can modulate oxidative stress damage in plants. However, the molecular mechanism controlling the expression of oxidative stress-response genes in NAG remains unknown. In Arabidopsis, exogenous treatment of acetic acid confers drought tolerance accompanied with the activation of some transcription factors including *ZAT10* through histone H4 acetylation (Kim et al., 2017). Therefore, we examined whether histone acetylation increased at *ZAT10* and *ZAT12* following NAG treatment. Chromatin immunoprecipitation – quantitative PCR (ChIP-qPCR) showed the levels of histone H4ac at the 5’ region of *ZAT10*, and *ZAT12* was increased in plants treated with NAG (**Figure 3A**). In contrast, histone H3 lysine 4 trimethylation (H3K4me3), whose modification levels are correlated with the activation of gene expression, was enhanced only at *ZAT12* and not at *ZAT10*, following NAG treatment (**Figure 3B**). The results suggest that NAG activates the expression of *ZAT10* and *ZAT12* by regulating histone acetylation.

**Figure 4:**
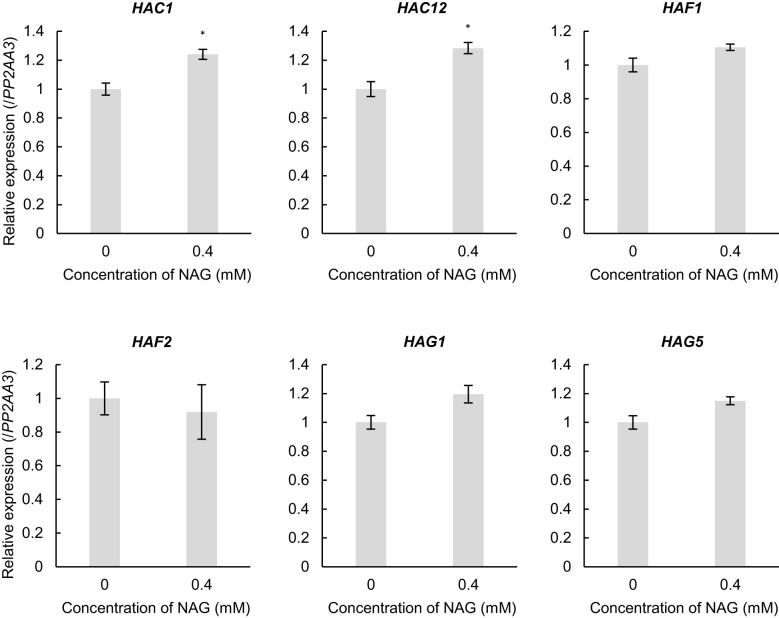
Induction of histone acetyltransferases with the treatment of N-acetylglutamic acid (NAG). Expression levels of *HAC1*, *HAC12*, *HAF1*, *HAF2*, *HAG1* and *HAG5* in seedlings treated with or without 0.4 mM NAG for 2 h. Error bars indicate the standard error. *n* = 3. *P < 0.05 (Student’s *t*-test).

To investigate the molecular mechanisms underlying histone acetylation of oxidative stress-response genes following NAG treatment, we investigated the expression levels of several histone acetyltransferases (HATs). Among the HATs, the expression levels of *ARABIDOPSIS HISTONE ACETYLTRANSFERASE OF THE CBP FAMILY 1* and *12* (*HAC1* and *HAC12*) were specifically increased in plants treated with NAG when compared with that in the control plants (**Figure 4**). Therefore, NAG facilitated *HAC1* and *HAC12* to activate the oxidative stress-response genes through histone acetylation.

### 3.4 N-acetylglutamic acid confers tolerance to oxidative stress in *Oryza sativa*

**Figure 5:**
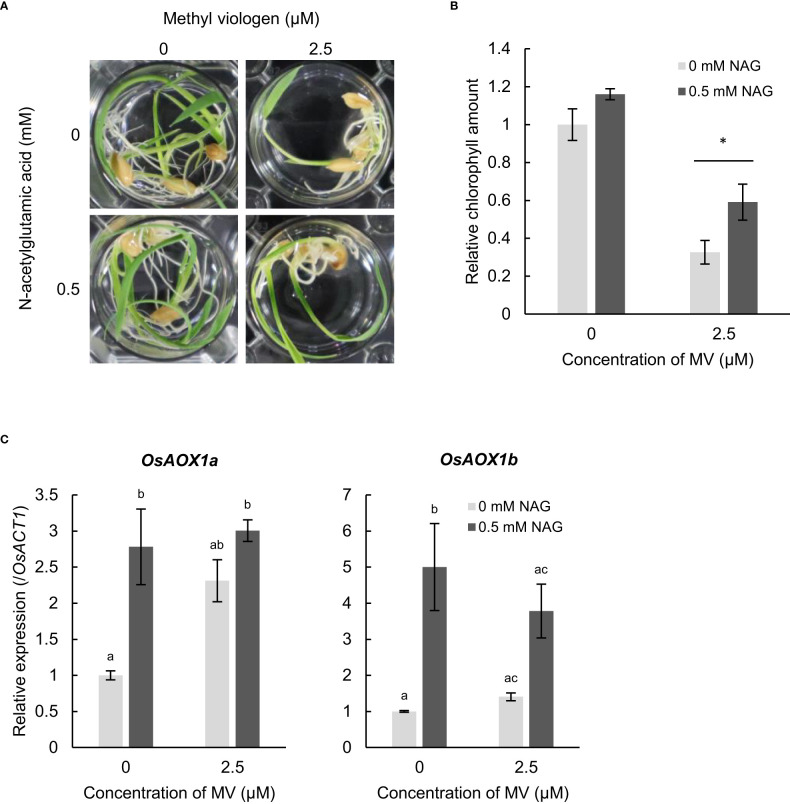
N-acetylglutamic acid (NAG) alleviates oxidative stress damage in *Oryza sativa*. **(A)** Seedlings treated with or without 0.5 mM NAG under 2.5 μM methyl viologen (MV) for 48 h. **(B)** Chlorophyll amounts in seedlings treated with or without 0.5 mM NAG under 2.5 μM MV for 48 h. Error bars indicate standard error. *n* = 15. *P < 0.05 (Tukey test). **(C)** Expression levels of *OsAOX1a* and *OsAOX1b* in seedlings treated with or without 0.4 mM NAG and 5 μM MV for 2 h. Error bars indicate the standard error. *n* = 3). *P* < 0.05 (Tukey test).

To examine whether NAG alleviates oxidative stress in plants other than Arabidopsis, we observed response to oxidative stress in the monocotyledonous plant rice. MV-induced chlorosis and reduced chlorophyll contents in the leaves of rice and Arabidopsis (**Figures 5A, B**). Following to MV, rice plants treated with NAG showed higher amounts of chlorophyll in the leaves than plants not treated with NAG (**Figures 5A, B**). Additionally, the expression levels of *OsAOX1a* and *OsAOX1b* increased in rice plants treated with NAG, which was similar to the results in Arabidopsis (**Figure 5C**). The results suggest that NAG can alleviate damage caused by oxidative stress by activating oxidative stress-response genes in rice.

## 4 Discussion

In this study, we demonstrated that NAG alleviates oxidative stress by regulating the expression of oxidative stress-response genes in Arabidopsis and rice. NAG is converted from Glu by N-acetylglutamate synthase (NAGS) in the first step of the arginine pathway in plants. *SlNAGS1* enhances tolerance to drought and salt stress in Arabidopsis, which is associated with the accumulation of ornithine, a precursor of arginine (Kalamaki et al., 2009). Overexpression of *Z. mays N-acetylglutamate kinase* (*ZmNAGK*), which phosphorylates NAG to proceed to arginine metabolism, in *Nicotiana tabacum*, resulted in greater resistance to drought stress than in wild-type plants with the accumulation of arginine, leading to the hypothesis that exogenous treatment with NAG modulates several environmental stress factors, including oxidative stress, with the activation of arginine metabolism as well as gene regulation activity involving stress-response genes (Liu et al., 2018). In response to heat stress, NO synthesised during arginine metabolism transduces the heat stress signal from the shoot apex to the roots to enhance systemic resistance by upregulating heat stress-response genes in Arabidopsis (He et al., 2022). Therefore, the role of NAG as an activator of systemic resistance via arginine metabolism in response to oxidative stress needs to be examined.

NAG is an intermediate converted from Glu during arginine metabolism in plants, suggesting that Glu-related compounds modulate the damage caused by environmental stress in plants. Exogenous treatment with Glu enhances systemic resistance in the immune system by upregulating defence-related genes, resulting in resistance to pathogen infection in Arabidopsis and rice (Kadotani et al., 2016; Goto et al., 2020). Additionally, deficiency in *GLUTAMATE DECARBOXYLASEs* (*GADs*), which synthesise GABA from Glu, results in a reduction in heat stress and high-light stress tolerance in Arabidopsis (Balfagón et al., 2022). Therefore, the application of Glu-related compounds, including NAG, may confer tolerance to several environmental stress factors in plants.

Exogenous treatment of NAG increased expression levels of oxidative stress-response genes in Arabidopsis (**Figure 2**). Overexpression of *ZAT10* or *ZAT12* in Arabidopsis induces the upregulation of genes involved in responses to some environmental stress factors, such as heat, oxidative, osmotic, and salinity stress, resulting in tolerance to the stress factors. Deficient mutant of *ZAT12* could not enhance the expression levels of *APX1* in response to oxidative stress, and *APX1* expression was activated with NAG treatment as well as *ZAT12* (**Figure 2**) (Rizhsky et al., 2004). Thus, transcriptional cascade from *ZAT12* to *APX1* may function in plants treated with NAG in response to oxidative stress. *ZAT10* directly regulates the transcriptional activity of genes related to the cadmium (Cd) uptake and detoxification to confer Cd tolerance under higher Cd conditions in Arabidopsis (Dang et al., 2022). Additionally, *ZAT12* prevents the overaccumulation of iron (Fe) in Arabidopsis shoots by negatively controlling the transcription involved in Fe uptake (Le et al., 2016). Therefore, NAG may alleviate environmental stress, excluding oxidative stress, through the regulation of *ZAT10* or *ZAT12* expression.

We also confirmed that histone levels H4ac were increased by NAG treatment (**Figure 3A**). Several synthetic and natural compounds can modulate the epigenetic modification of stress-response genes, resulting in stress tolerance in plants. Treatment with Ky-2, an inhibitor of HDAC, alleviates salinity stress through the activation of the Na^+^/H^+^ antiporter *SOS1* based on the upregulation of histone H4ac in Arabidopsis (Sako et al., 2016). The induction of JA signalling associated with the hyperacetylation of histones H3 and H4 via the HDAC inhibitor suberoylanilde hydroxamic acid (SAHA) confers salinity stress tolerance in *Manihot esculenta*. Acetic acid activates jasmonate signalling associated with an increase in histone H4ac levels, resulting in drought stress tolerance in Arabidopsis, rice, *Z. mays*, *T. aestivum*, and *B. napus* (Kim et al., 2017). Radioisotope experiments using acetic acid labelled with ^14^C show the possibility that acetic acid is used as a substrate for histone H4ac in Arabidopsis. Interestingly, acetic acid is also used as a substrate for acetylation of tRNA catalysed by acetyltransferase *tmcAL* in *Bacillus subtilis*, suggesting that NAG might function as a substrate of acetyl group for acetylation at oxidative stress-responsive genes, including histone acetyltransferases (Taniguchi et al., 2018).

Although several studies have explored chemical compounds that alleviate the damage caused by environmental stresses based on histone acetylation, little is known about the effects of chemical compounds involved in epigenetic modification other than histone acetylation. Studies using specific Arabidopsis mutants have demonstrated that histone methylation contributes to stress tolerance. For example, deficiency in *LDL1*, which is a histone demethylase of histone H3 Lys 4 di-methylation (H3K4me2), causes the accumulation of DNA damage due to genotoxic stress in Arabidopsis (Hirakawa et al., 2019). Additionally, the removal of histone H3 Lys 27 tri-methylation (H3K27me3) on heat stress-response genes by the histone demethylase *JUMONJIs* (*JMJ26, JMJ29*, *ERF6*, *REF6*) is required for priming against heat stress, indicating that manipulation of histone methylation using chemical compounds could enhance stress tolerance (Yamaguchi et al., 2021).

Although exogenous treatment with NAG specifically increases the expression levels of the histone acetyltransferases *HAC1* and *HAC12*, which are involved in the regulation of flowering time and sugar response, the molecular mechanisms underlying the upregulation of the genes by NAG remains unknown (**Figure 4**) (Han et al., 2007; Chen et al., 2019). Recently, it has been revealed that defence responses attributed to volatile organic compounds (VOCs) emitted from plants in response to herbivory could be enhanced based on chromatin remodelling (Onosato et al., 2022). Among VOCs, caryophyllene, a sesquiterpene, increases the expression levels of defence-related genes in tobacco BY-2 cells through binding with TOPLESS (TPL)-like protein, which functions as a transcriptional co-repressor by interacting with the transcription factor (Nagashima et al., 2019). The findings of the present study indicate that NAG could activate the expression of oxidative stress-response genes, including histone acetyltransferases, based on chromatin remodelling activities modulated by chromatin modifiers such as TPL-like proteins.

## Data availability statement

The raw data supporting the conclusions of this article will be made available by the authors, without undue reservation.

## Author contributions

TH designed the study. TH and ST conducted the experiments. TH analysed the data. TH and KO wrote the manuscript. All authors contributed to the article and approved the submitted version.

## Supplementary material

Supplementary file S1 List of primers used in this study

## References

[B1] Alscher R. G. Erturk N. Heath L. S. (2002). Role of superoxide dismutases (SODs) in controlling oxidative stress in plants. J. Exp. Bot. 53, 1331–1341. doi: 10.1093/jexbot/53.372.1331 11997379

[B2] Anjum N. A. Sharma P. Gill S. S. Hasanuzzaman M. Khan E. A. Kachhap K.. (2016). Catalase and ascorbate peroxidase-representative H2O2-detoxifying heme enzymes in plants. Environ. Sci. pollut. Res. Int. 23, 19002–19029. doi: 10.1007/s11356-016-7309-6 27549233

[B3] Balfagón D. Gómez-Cadenas A. Rambla J. L. Granell A. de Ollas C. Bassham D. C.. (2022). γ-aminobutyric acid plays a key role in plant acclimation to a combination of high light and heat stress. Plant Physiol. 188, 2026–2038. doi: 10.1093/plphys/kiac010 35078231 PMC8968390

[B4] Baxter A. Mittler R. Suzuki N. (2014). ROS as key players in plant stress signalling. J. Exp. Bot. 65, 1229–1240. doi: 10.1093/jxb/ert375 24253197

[B5] Chen Q. Xu X. Xu D. Zhang H. Zhang C. Li G. (2019). WRKY18 and WRKY53 coordinate with histone ACETYLTRANSFERASE1 to regulate rapid responses to sugar. Plant Physiol. 180, 2212–2226. doi: 10.1104/pp.19.00511 31182557 PMC6670108

[B6] Dang F. Li Y. Wang Y. Lin J. Du S. Liao X. (2022). ZAT10 plays dual roles in cadmium uptake and detoxification in arabidopsis. Front. Plant Sci. 13. doi: 10.3389/fpls.2022.994100 PMC9468636 36110357

[B7] Gechev T. S. Van Breusegem F. Stone J. M. Denev I. Laloi C. (2006). Reactive oxygen species as signals that modulate plant stress responses and programmed cell death. BioEssays 28, 1091–1101. doi: 10.1002/bies.20493 17041898

[B8] Goto Y. Maki N. Ichihashi Y. Kitazawa D. Igarashi D. Kadota Y.. (2020). Exogenous treatment with glutamate induces immune responses in arabidopsis. Mol. Plant Microbe Interact. 33, 474–487. doi: 10.1094/MPMI-09-19-0262-R 31721650

[B9] Han S. K. Song J. D. Noh Y. S. Noh B. (2007). Role of plant CBP/p300-like genes in the regulation of flowering time. Plant J. 49, 103–114. doi: 10.1111/j.1365-313X.2006.02939.x 17144897

[B10] He N. Y. Chen L. S. Sun A. Z. Zhao Y. Yin S. N. Guo F. Q. (2022). A nitric oxide burst at the shoot apex triggers a heat-responsive pathway in arabidopsis. Nat. Plants 8, 434–450. doi: 10.1038/s41477-022-01135-9 35437002

[B11] Hirakawa T. Kuwata K. Gallego M. E. White C. I. Nomoto M. Tada Y.. (2019). LSD1-LIKE1-mediated H3K4me2 demethylation is required for homologous recombination repair. Plant Physiol. 181, 499–509. doi: 10.1104/pp.19.00530 31366719 PMC6776857

[B12] Kadotani N. Akagi A. Takatsuji H. Miwa T. Igarashi D. (2016). Exogenous proteinogenic amino acids induce systemic resistance in rice. BMC Plant Biol. 16, 60. doi: 10.1186/s12870-016-0748-x 26940322 PMC4778346

[B13] Kalamaki M. S. Alexandrou D. Lazari D. Merkouropoulos G. Fotopoulos V. Pateraki I.. (2009). Over-expression of a tomato n-acetyl-L-glutamate synthase gene (SlNAGS1) in arabidopsis thaliana results in high ornithine levels and increased tolerance in salt and drought stresses. J. Exp. Bot. 60, 1859–1871. doi: 10.1093/jxb/erp072 19357433 PMC2671631

[B14] Kim D. S. Hwang B. K. (2014). An important role of the pepper phenylalanine ammonia-lyase gene (PAL1) in salicylic acid-dependent signalling of the defence response to microbial pathogens. J. Exp. Bot. 65, 2295–2306. doi: 10.1093/jxb/eru109 24642849 PMC4036500

[B15] Kim J. M. To T. K. Matsui A. Tanoi K. Kobayashi N. I. Matsuda F.. (2017). Acetate-mediated novel survival strategy against drought in plants. Nat. Plants 3, 17097. doi: 10.1038/nplants.2017.97 28650429

[B16] Kurita K. Sakamoto T. Yagi N. Sakamoto Y. Ito A. Nishino N.. (2017). Live imaging of H3K9 acetylation in plant cells. Sci. Rep. 7, 45894. doi: 10.1038/srep45894 28418019 PMC5394682

[B17] Le C. T. T. Brumbarova T. Ivanov R. Stoof C. Weber E. Mohrbacher J.. (2016). ZINC FINGER OF arabidopsis THALIANA12 (ZAT12) interacts with FER-LIKE IRON DEFICIENCY-INDUCED transcription factor (FIT) linking iron deficiency and oxidative stress responses. Plant Physiol. 170, 540–557. doi: 10.1104/pp.15.01589 26556796 PMC4704599

[B18] Li Y.-J. Hai R.-L. Du X.-H. Jiang X.-N. Lu H. (2009). Over-expression of a populus peroxisomal ascorbate peroxidase (PpAPX) gene in tobacco plants enhances stress tolerance. Plant Breed. 128, 404–410. doi: 10.1111/j.1439-0523.2008.01593.x

[B19] Liu W. Xiang Y. Zhang X. Han G. Sun X. Sheng Y.. (2018). Over-expression of a maize n-acetylglutamate kinase gene (ZmNAGK) improves drought tolerance in tobacco. Front. Plant Sci. 9. doi: 10.3389/fpls.2018.01902 PMC6328498 30662448

[B20] Matsui A. Todaka D. Tanaka M. Mizunashi K. Takahashi S. Sunaoshi Y.. (2022). Ethanol induces heat tolerance in plants by stimulating unfolded protein response. Plant Mol. Biol. 110, 131–145. doi: 10.1007/s11103-022-01291-8 35729482

[B21] Nagashima A. Higaki T. Koeduka T. Ishigami K. Hosokawa S. Watanabe H.. (2019). Transcriptional regulators involved in responses to volatile organic compounds in plants. J. Biol. Chem. 294, 2256–2266. doi: 10.1074/jbc.RA118.005843 30593507 PMC6378981

[B22] Nakabayashi R. Yonekura-Sakakibara K. Urano K. Suzuki M. Yamada Y. Nishizawa T.. (2014). Enhancement of oxidative and drought tolerance in arabidopsis by overaccumulation of antioxidant flavonoids. Plant J. 77, 367–379. doi: 10.1111/tpj.12388 24274116 PMC4282528

[B23] Nishimura A. Kawahara N. Takagi H. (2013). The flavoprotein Tah18-dependent NO synthesis confers high-temperature stress tolerance on yeast cells. Biochem. Biophys. Res. Commun. 430, 137–143. doi: 10.1016/j.bbrc.2012.11.023 23159617

[B24] Nishimura A. Kotani T. Sasano Y. Takagi H. (2010). An antioxidative mechanism mediated by the yeast n-acetyltransferase Mpr1: oxidative stress-induced arginine synthesis and its physiological role. FEMS Yeast Res. 10, 687–698. doi: 10.1111/j.1567-1364.2010.00650.x 20550582

[B25] Niu Y. Bai J. Liu X. Zhang H. Bao J. Zhao W.. (2022). Histone deacetylase 9 transduces heat signal in plant cells. Proc. Natl. Acad. Sci. U. S. A. 119, e2206846119. doi: 10.1073/pnas.2206846119 36322735 PMC9661188

[B26] Onosato H. Fujimoto G. Higami T. Sakamoto T. Yamada A. Suzuki T.. (2022). Sustained defense response via volatile signaling and its epigenetic transcriptional regulation. Plant Physiol. 189, 922–933. doi: 10.1093/plphys/kiac077 35201346 PMC9157098

[B27] Quan J. Zheng W. Wu M. Shen Z. Tan J. Li Z.. (2022). Glycine betaine and β-aminobutyric acid mitigate the detrimental effects of heat stress on Chinese cabbage (Brassica rapa l. ssp. pekinensis) seedlings with improved photosynthetic performance and antioxidant system. Plants (Basel) 11, 1213. doi: 10.3390/plants11091213 35567214 PMC9105105

[B28] Rizhsky L. Davletova S. Liang H. Mittler R. (2004). The zinc finger protein Zat12 is required for cytosolic ascorbate peroxidase 1 expression during oxidative stress in arabidopsis. J. Biol. Chem. 279, 11736–11743. doi: 10.1074/jbc.M313350200 14722088

[B29] Sako K. Kim J. M. Matsui A. Nakamura K. Tanaka M. Kobayashi M.. (2016). Ky-2, a histone deacetylase inhibitor, enhances high-salinity stress tolerance in arabidopsis thaliana. Plant Cell Physiol. 57, 776–783. doi: 10.1093/pcp/pcv199 26657894

[B30] Sako K. Nguyen H. M. Seki M. (2021). Advances in chemical priming to enhance abiotic stress tolerance in plants. Plant Cell Physiol. 61, 1995–2003. doi: 10.1093/pcp/pcaa119 32966567

[B31] Shigeoka S. Ishikawa T. Tamoi M. Miyagawa Y. Takeda T. Yabuta Y.. (2002). Regulation and function of ascorbate peroxidase isoenzymes. J. Exp. Bot. 53, 1305–1319. doi: 10.1093/jexbot/53.372.1305 11997377

[B32] Sokol A. Kwiatkowska A. Jerzmanowski A. Prymakowska-Bosak M. (2007). Up-regulation of stress-inducible genes in tobacco and arabidopsis cells in response to abiotic stresses and ABA treatment correlates with dynamic changes in histone H3 and H4 modifications. Planta 227, 245–254. doi: 10.1007/s00425-007-0612-1 17721787

[B33] Taniguchi T. Miyauchi K. Sakaguchi Y. Yamashita S. Soma A. Tomita K.. (2018). Acetate-dependent tRNA acetylation required for decoding fidelity in protein synthesis. Nat. Chem. Biol. 14, 1010–1020. doi: 10.1038/s41589-018-0119-z 30150682

[B34] Yamaguchi N. Matsubara S. Yoshimizu K. Seki M. Hamada K. Kamitani M.. (2021). H3K27me3 demethylases alter HSP22 and HSP17.6C expression in response to recurring heat in arabidopsis. Nat. Commun. 12, 3480. doi: 10.1038/s41467-021-23766-w 34108473 PMC8190089

[B35] Yamaguchi N. Winter C. M. Wu M. F. Kwon C. S. William D. A. Wagner D. (2014). PROTOCOLS: chromatin immunoprecipitation from arabidopsis tissues. Arabidopsis Book 12, e0170. doi: 10.1199/tab.0170 24653666 PMC3952383

[B36] Yan H. Li Q. Park S. C. Wang X. Liu Y. J. Zhang Y. G.. (2016). Overexpression of CuZnSOD and APX enhance salt stress tolerance in sweet potato. Plant Physiol. Biochem. 109, 20–27. doi: 10.1016/j.plaphy.2016.09.003 27620271

[B37] Yang C. Li W. Cao J. Meng F. Yu Y. Huang J.. (2017). Activation of ethylene signaling pathways enhances disease resistance by regulating ROS and phytoalexin production in rice. Plant J. 89, 338–353. doi: 10.1111/tpj.13388 27701783

[B38] Zimmerli L. Hou B. H. Tsai C. H. Jakab G. Mauch-Mani B. Somerville S. (2008). The xenobiotic beta-aminobutyric acid enhances arabidopsis thermotolerance. Plant J. 53, 144–156. doi: 10.1111/j.1365-313X.2007.03343.x 18047473

